# Research on Alternating Current Field Measurement Method for Buried Defects of Titanium Alloy Aircraft Skin

**DOI:** 10.3390/s24041347

**Published:** 2024-02-19

**Authors:** Chunhui Liao, Ruize Wang, Cheng Lv, Tao Chen, Zhiyang Deng, Xiaochun Song

**Affiliations:** 1 Hubei Key Laboratory of Modern Manufacturing Quantity Engineering, School of Mechanical Engineering, Hubei University of Technology, Wuhan 430068, China; 102110138@hbut.edu.cn (R.W.); chentao@hnu.edu.cn (T.C.); dzy@hust.edu.cn (Z.D.); songxc@mail.hbut.edu.cn (X.S.); 2 Hubei Special Equipment Inspection Testing Institute, Wuhan 430077, China; chenlv_nick@163.com

**Keywords:** alternating current field measurement, titanium alloy, buried defects, characteristic signals, aircraft skin, probe optimization

## Abstract

Titanium alloys are extensively used in the manufacturing of key components in aerospace engines and aircraft structures due to their excellent properties. However, aircraft skins in harsh operating environments are subjected to long-term corrosion and pressure concentrations, which can lead to the formation of cracks and other defects. In this paper, a detection probe is designed based on the principle of alternating current field measurement, which can effectively detect both surface and buried defects in thin-walled titanium alloy plates. A finite element simulation model of alternating current field measurement detection for buried defects in thin-walled TC4 titanium alloy plates is established using COMSOL 5.6 software. The influence of defect length, depth, and excitation frequency on the characteristic signals is investigated, and the detection probe is optimized. Simulation and experimental results demonstrate that the proposed detection probe exhibits high detection sensitivity to varying lengths and depths of buried defects, and can detect small cracks with a length of 3 mm and a burial depth of 2 mm, as well as deep defects with a length of 10 mm and a burial depth of 4 mm. The feasibility of this probe for detecting buried defects in titanium alloy aircraft skin is confirmed.

## 1. Introduction

Titanium alloy TC4 is a metal alloy composed of titanium and other metals, known for its high strength, corrosion resistance, and heat resistance. It is widely used in aerospace, shipbuilding, chemical, and other fields [1,2,3]. With the increasing demands in national defense equipment and technological advancements, titanium alloys are being used more extensively in the manufacturing of key components in aerospace engines and aircraft structures, such as fuselage skins, engine fans, blades, and fasteners. However, aircraft skin in service due to corrosion and stress concentration and other reasons will produce cracks, dents, corrosion holes, and other defects, which results in a reduction in the skin’s strength and a decline in its bearing capacity. If these defects are not detected in a timely and accurate manner, they can continuously grow and seriously threaten flight safety. Therefore, it is crucial to detect defects promptly to prevent accidents.

Currently, commonly used non-destructive testing techniques for titanium alloy defect detection include ultrasonic testing, eddy current testing, radiographic testing, and infrared thermography. However, these methods have their limitations. The coarse α-phase of titanium alloys significantly reduces the signal-to-noise ratio in ultrasonic testing, and conventional ultrasonic testing has low precision, shallow detection depth, and cannot effectively detect buried defects. Additionally, if the wall thickness of the tested workpiece is too thin, it will cause temporal overlap of the backscattered echoes, making it difficult to calculate the time difference of the echoes [4,5]. Eddy current testing is mainly used for detecting surface and near-surface defects due to the skin effect and is not effective in detecting buried defects. Eddy current probes are also highly sensitive to lift-off height. Defect signals are easily submerged in the large baseline caused by the background magnetic field [6,7,8]. Radiographic testing may miss defects due to factors such as edge effects and certain limitations in detecting titanium alloy specimens, as they have a low X-ray absorption coefficient and exhibit some degree of attenuation. Radiographic testing is also associated with radioactive contamination [9,10]. Ultrasonic infrared thermography relies on heating the area of the defect to obtain an infrared thermal image, but the high stiffness of titanium alloys makes it difficult to achieve the required excitation energy for this method [11,12,13,14].

The above-mentioned testing methods all have their limitations. To address the issue of buried defects in titanium alloy aircraft skin, the Alternating Current Field Measurement (ACFM) technique has emerged as a new non-destructive testing technology based on the principle of electromagnetic induction. The ACFM method generates an eddy current field distributed along the surface of the workpiece, with the induced current passing through the defect. Within a certain range of lift-off, changes in the probe’s lift-off height have a minimal impact on the eddy current strength. ACFM offers advantages such as non-contact inspection, insensitivity to lift-off, qualitative and quantitative capabilities, and good penetration through non-metallic coatings [15,16,17]. Furthermore, this detection method is suitable for both ferromagnetic and non-ferromagnetic metallic materials. ACFM has been widely applied in various fields. For example, Feng et al. [18] designed an ACFM probe for the quantitative evaluation of axial cracks on the inner surface of long-distance pipelines caused by corrosion. Zhao et al. [19] designed a flexible array ACFM probe suitable for drilling pipes with different diameters and capable of effectively detecting internal and external cracks in pipes. Muñoz et al. [20] successfully improved the reliability of detection results for surface fracture defects on steel rails by applying a non-uniform spline approximation algorithm to the data collected by ACFM probes. Currently, the ACFM method is still mainly applied to the detection of surface and near-surface defects, and there are still many problems to be solved in the detection of deeper buried defects.

In this study, based on the basic principle of ACFM detection, a finite element simulation model for ACFM detection of buried defects in thin-walled titanium alloy plates is established. The influence of defect length, depth, and excitation frequency on the characteristic magnetic field signals (Bx and Bz) is investigated. An ACFM detection probe is designed and an ACFM system is constructed. The experimental results demonstrate that the ACFM probe can effectively overcome the lift-off effect and exhibit high detection sensitivity to varying lengths and depths of buried defects. The experimental results are in good agreement with the simulation results. The ACFM probe is capable of detecting small cracks with a length of 3 mm and a burial depth of 2 mm as well as deep defects with a length of 10 mm and a burial depth of 4 mm. The ACFM probe shows good detection capabilities for buried defects in titanium alloy plates.

## 2. Basic Principles of ACFM

**Figure 1:**
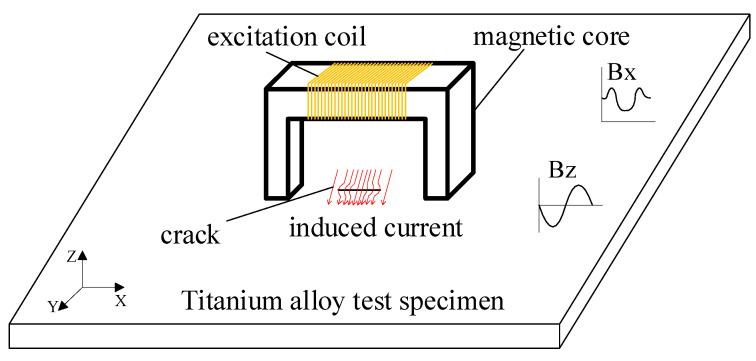
Schematic diagram of ACFM detection principle.

The principle of the ACFM detection technique is illustrated in Figure 1. An alternating current is applied to the coil, inducing eddy currents on the surface of the test specimen. If there are no cracks on the surface, the induced eddy currents will distribute uniformly. However, if there are cracks on the surface, the eddy currents will accumulate around the crack, causing distortion in the spatial magnetic field near the defect. The magnetic field components Bx and Bz are extracted by the detection coil, and these components can be used to infer information about the size of surface defects [21,22]. The Bx signal exhibits a characteristic with two peaks and one trough between them, which can indicate the depth information of the surface defect. The Bz signal exhibits a characteristic with one peak and one trough, which can indicate the length of the surface defect.

## 3. Finite Element Simulation

Due to the skin effect, the ACFM technique is currently mainly used for surface and near-surface defect detection. However, at the right excitation frequency and material properties, induced currents can penetrate into the deeper layers of the material and accumulate in the region of buried defects. In this section, we investigate the distribution characteristics of induced currents in the region of buried defects, the characteristics of magnetic field signals, and the influence of defect length, depth, and excitation frequency on the magnetic field signals.

### 3.1. Simulation Analysis of Buried Defects

According to the formula for skin depth (1), the penetration depth (*δ*) is related to the relative magnetic permeability (*μr*), electrical conductivity (*σ*), and excitation frequency (*f*) of the test specimen. When other conditions are constant, a lower excitation frequency results in a deeper penetration depth (*δ*) for the induced currents. The thickness of the general aircraft skin is 2~5 mm. For TC4 titanium alloy with a relative magnetic permeability of 1 and an electrical conductivity (*σ*) of 6 × 10^5^ S/m, at an excitation frequency (*f*) of 10 kHz, the penetration depth (*δ*) is 6.54 mm. At this frequency, the induced current can completely penetrate a 6 mm thick titanium alloy plate.

(1) $$\delta =\frac{1}{\sqrt{\pi f\mu_{r}\mu_{o}\sigma }}$$

**Figure 2:**
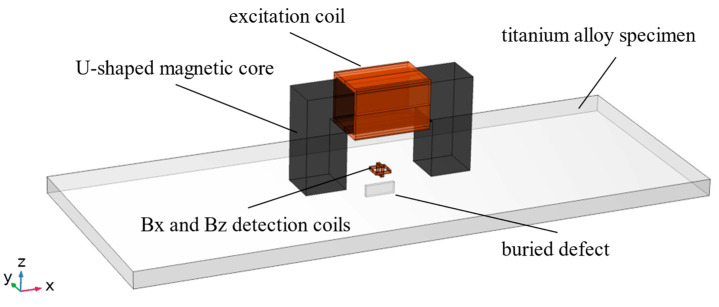
ACFM simulation model of buried defect in titanium alloy plate.

Using COMSOL Multiphysics 5.6 finite element simulation software, a simulation model for ACFM detection of buried defects in titanium alloy plates was established, as shown in Figure 2. The simulation model consists of a titanium alloy specimen, a U-shaped magnetic core, an excitation coil, and two detection coils. In order to ensure the consistency between simulation and experiment, the size of the U-shaped core set in simulation is the same as the size of the inspection probe fabricated later, and the size of the buried defects set in simulation is the same as the defects processed in the experimental specimen. The dimensions and material parameters of the simulation model are shown in Table 1 and Table 2.

**Figure 3:**
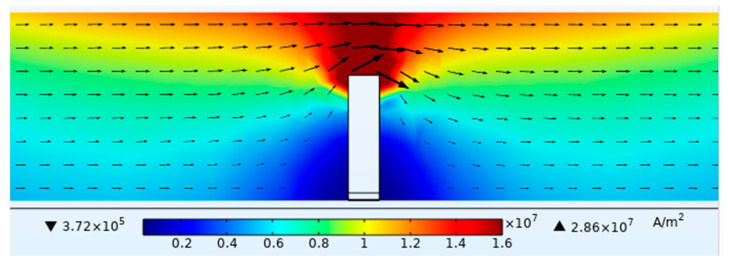
YZ plane induced current distribution of buried defects.

**Figure 4:**
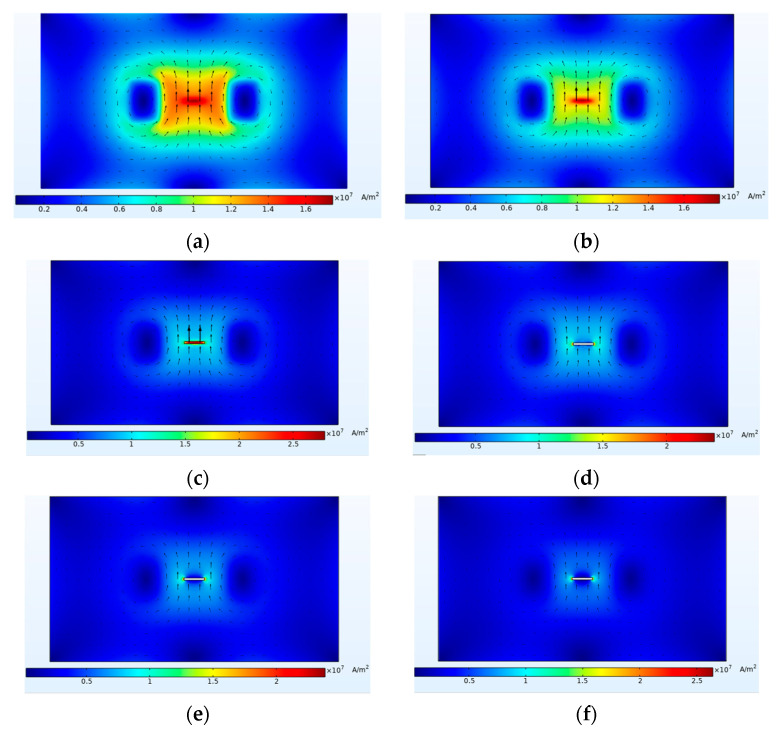
XY plane induced current distribution at different sections. (**a**) Section at 0 mm. (**b**) Section at −1 mm. (**c**) Section at −2 mm. (**d**) Section at −3 mm. (**e**) Section at −4 mm. (**f**) Section at −5 mm.

The excitation frequency was set to 10 kHz, and the driving current was 1A. The depth of the defect in the titanium alloy plate was set to 4 mm, corresponding to a burial depth of 2 mm, and the lift-off height of the probe was set to 1 mm. The distribution of induced currents in the YZ plane of the buried defect was obtained from the simulation, as shown in Figure 3. The distribution of induced currents in different depths of the XY plane was captured and presented in Figure 4. As the depth of the XY plane increased, the intensity of the induced currents decreased, and the currents bypassed the buried defect and accumulated near it, resulting in distortion in the spatial magnetic field near the defect.

**Figure 5:**
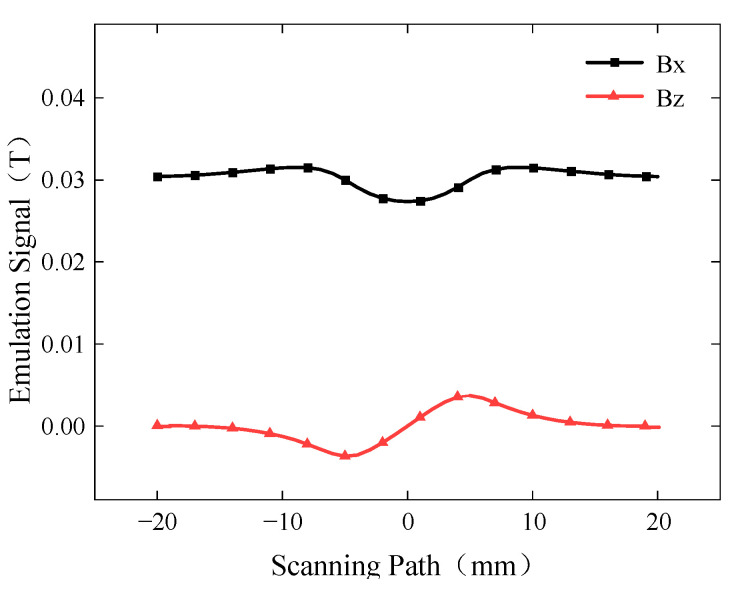
Magnetic field signals of buried defects in ACFM.

The extracted Bx and Bz signals of the buried defect are shown in Figure 5. The Bx signal exhibits the characteristic of having two peaks with a trough in between, while the Bz signal exhibits the characteristic of having one peak and one trough. The signal pattern of buried defects is initially consistent with that of surface defects, and the characteristic signal pattern of buried defects will be comprehensively explored subsequently in terms of several influencing factors, including defect length, depth, and excitation frequency.

The sensitivity of the probe is an important indicator of its detection performance. For analysis purposes, the detection sensitivity of the *Bx* signal (*Sx*) and the peak-to-peak value of the *Bz* signal (∆*Bz*) were defined. The specific calculation formulas are as follows:

(2) $$Sx=\frac{Bx_{\max}-Bx_{\min}}{Bx_{0}}$$

(3) $$\Delta Bz=Bz_{\max}-Bz_{\min}$$

In Formulas (2) and (3), *Bx_max_* and *Bx_min_* are the maximum and the minimum values of Bx when a defect is present, *Bx*_0_ is the background magnetic field of *Bx* when no defect is present. *Bz_min_* and *Bz_max_* represent the minimum and maximum values of *Bz* when a defect is present, respectively. A higher value of *Sx* and ∆*Bz* indicates better detection sensitivity of the probe.

### 3.2. Influence of Burial Depth on Detection Results

**Figure 6:**
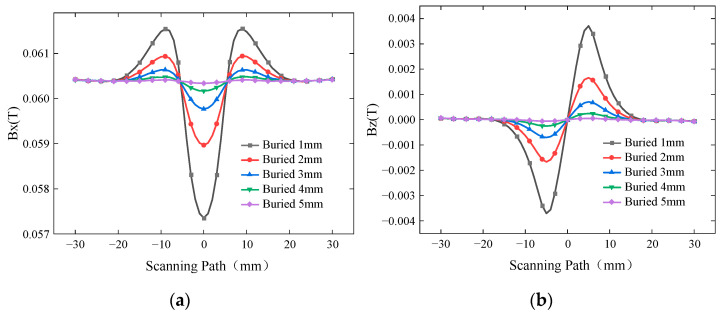
*Bx* and *Bz* signals at different burial depths. (**a**) *Bx* signals at different burial depths. (**b**) *Bz* signals at different burial depths.

**Figure 7:**
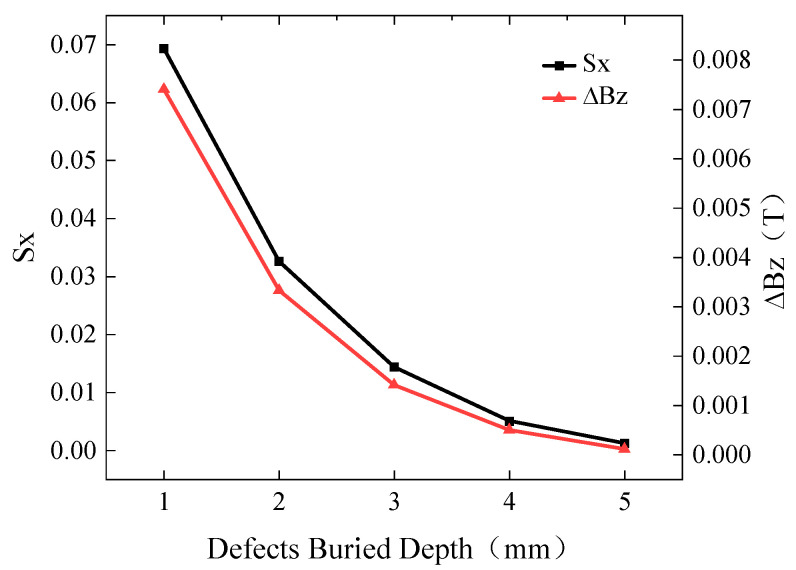
Variation in *Sx* and ∆*Bz* at different burial depths.

To investigate the influence of burial depth on the characteristic signals, a buried defect of length 10 mm and width 0.5 mm was simulated with burial depths ranging from 1 mm to 5 mm. The excitation frequency was set to 10 kHz, and the driving current was 1A. The simulated *Bx* and *Bz* signals are shown in Figure 6, while the curves of *Sx* and ∆*Bz* with respect to burial depth are presented in Figure 7.

The *Bx* signal exhibits two peaks with a trough in between, while the *Bz* signal exhibits one peak and one trough. As the burial depth increases, the induced currents gradually decay and become more sparse, resulting in smaller magnetic field signals. Hence, both *Sx* and ∆*Bz* decrease with increasing burial depth.

### 3.3. Influence of Buried Defect Length on Detection Results

**Figure 8:**
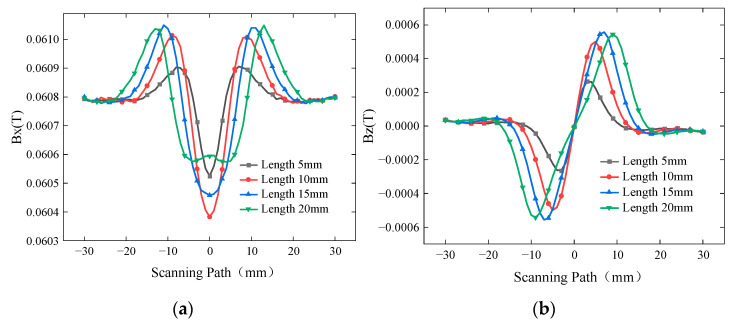
*Bx* and *Bz* signals for buried defects of different lengths. (**a**) *Bx* signals for buried defects of different lengths. (**b**) *Bz* signals for buried defects of different lengths.

**Figure 9:**
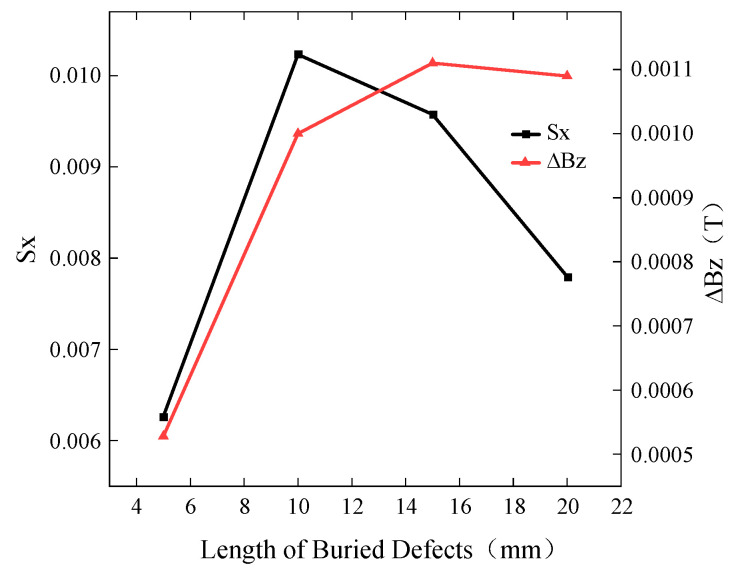
Variation in *Sx* and ∆*Bz* for buried defects of different lengths.

To investigate the influence of buried defect length on the detection results, a defect with a width of 0.5 mm and a burial depth of 2 mm was simulated with lengths of 5 mm, 10 mm, 15 mm, and 20 mm. The excitation frequency was set to 10 kHz, and the driving current was 1A. The simulated *Bx* and *Bz* signals are shown in Figure 8, while the curves of *Sx* and ∆*Bz* with respect to defect length are presented in Figure 9.

As the length of the defect continues to increase, the induced current density around the defect decreases, resulting in a weakening of the induced current’s strength. Therefore, when the defect length exceeds a certain range, both *Sx* and ∆*Bz* show a trend of initially increasing and then decreasing. When the defect length reaches 20 mm, the induced current density at the center of the defect decreases, causing the *Bx* signal trough to become raised. *Sx* increases for defect lengths ranging from 5 mm to 10 mm. However, *Sx* decreases for defect lengths ranging from 10 mm to 20 mm. The spacing between the peaks and troughs of the *Bz* signal corresponds to the defect length set in the simulation. Thus, the *Bz* signal can characterize the length of buried defects. The ∆*Bz* increases for defect lengths ranging from 5 mm to 15 mm. But it decreases for defect lengths ranging from 15 mm to 20 mm.

### 3.4. Influence of Excitation Frequency on Detection Results

**Figure 10:**
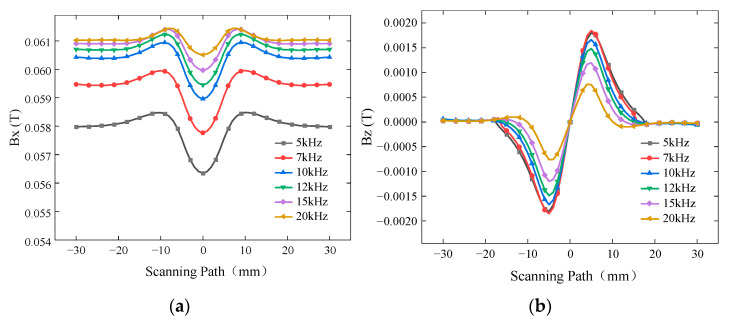
*Bx* and *Bz* signals for different excitation frequencies. (**a**) *Bx* signals for buried defects with different excitation frequencies. (**b**) *Bz* signals for buried defects with different excitation frequencies.

**Figure 11:**
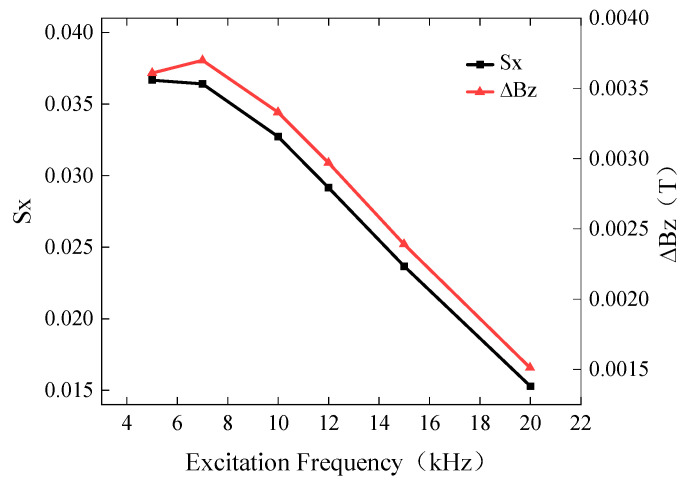
Variation in *Sx* and ∆*Bz* for different excitation frequencies.

From Formula (1), it is known that the excitation frequency significantly affects the penetration depth and, thus, the detection signal. To investigate the influence of excitation frequency on the detection results, a defect with a length of 10 mm, a width of 0.5 mm, and a burial depth of 2 mm was simulated. The excitation frequencies were set to 5 kHz, 7 kHz, 10 kHz, 12 kHz, 15 kHz, and 20 kHz, while the driving current was 1A. The simulated *Bx* and *Bz* signals are shown in Figure 10, while the curves of *Sx* and ∆*Bz* with respect to excitation frequency are presented in Figure 11.

The background magnetic field intensity of the Bx signal increases with increasing frequency. In the frequency range of 5 kHz to 20 kHz, *Sx* decreases. Similarly, ∆*Bz* increases slowly in the frequency range of 5 kHz to 7 kHz, but it sharply decreases in the frequency range of 7 kHz to 20 kHz. Therefore, the probe exhibits optimal detection sensitivity at an excitation frequency of 7 kHz in the simulation. The optimum excitation frequency for actual testing needs to be determined by field commissioning.

## 4. ACFM System Design

### 4.1. Specimen Preparation

**Figure 12:**
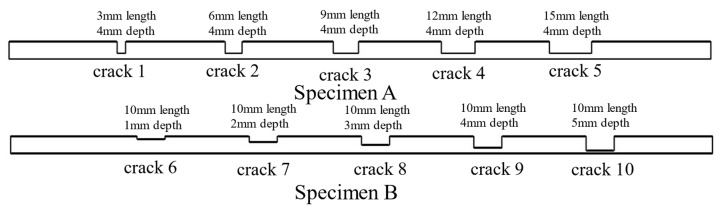
Schematic of titanium alloy plate defect parameters and numbers.

To investigate the influence of buried defect length and depth on the characteristic signals under the ACFM method, 10 rectangular groove defects with a width of 0.5 mm were machined on two titanium alloy specimens, each having a thickness of 6 mm, using an EDM-NC wire-cut machine. In this paper, the first priority is to realize the detection study of regularly buried defects, and the subsequent work will focus on the study of composite and irregular types of buried defects. The depths of the five defects in specimen A were all 4 mm, while the lengths of the five defects in specimen B were all 10 mm. The detailed parameters of the defects and their corresponding numbering are shown in Figure 12.

### 4.2. Probe Design

Optimizing the detection probe is crucial because the signal accuracy of buried defects is lower than that of surface defects. Factors affecting detection signal sensitivity include the size, shape, and number of turns of the detection probe, as well as sensor selection.

**Figure 13:**
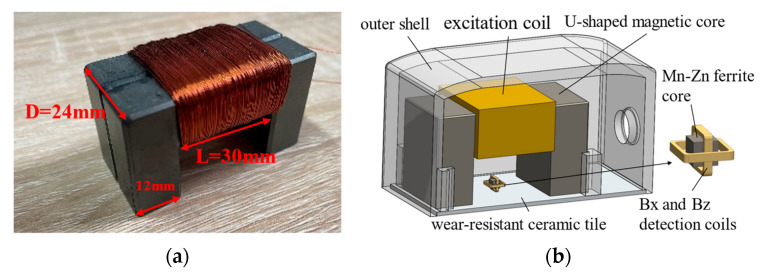
Diagram of ACFM detection probe. (**a**) Schematic diagram of U-shaped magnetic core dimensions. (**b**) 3D model of ACFM detection probe.

The dimensions of the U-shaped magnetic core are shown in Figure 13a. The designed probe, as shown in Figure 13b, consists of a U-shaped magnetic core, an excitation coil, two coils for sensing X and Z field components, a detection coil magnetic core, wear-resistant ceramic tiles, and an outer shell. The U-shaped and detection coil magnetic cores are made of manganese-zinc ferrite, the physical parameters of which are listed in Table 2. The sensing sensors, used for signal acquisition, are made of copper wire. This type of sensor has a large measurement range and is less affected by residual magnetism [23].

#### 4.2.1. Simulation of Excitation Coil Turns

Based on the detection principle of the ACFM method, the magnetic field generated by the excitation coil is guided through the U-shaped magnetic core to the surface of the workpiece, creating a uniform induced current perpendicular to the defect on the workpiece surface. The detection coil picks up the distorted magnetic field signal. Therefore, the larger and stronger the excitation field generated by the excitation coil, the better the detection performance of the probe. According to Formula (4), the magnetic induction intensity (*B*) generated by the excitation coil is dependent on factors such as the number of turns (*N*) of the coil. Here, *U* represents the power supply voltage, *R* represents the wire resistance, *μ*_0_ represents the vacuum permeability, and *δ* represents the air gap length.

(4) $$B=\frac{NU}{R\delta }\mu_{0}=\frac{NI}{\delta }\mu_{0}$$

**Figure 14:**
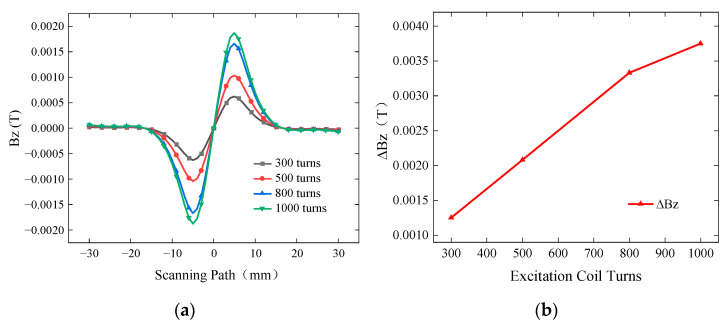
*Bz* signal and ∆*Bz* for different excitation coil turns. (**a**) *Bz* signals for buried defects with different excitation coil turns. (**b**) Variation in ∆*Bz* for different excitation coil turns.

To improve the detection sensitivity of the probe, the simulation was set up for excitation coil turns of 300, 500, 800, and 1000. A defect with a length of 10 mm, a width of 0.5 mm, and a burial depth of 2 mm was simulated. The excitation frequency was set to 7 kHz, and the driving current was 1A. The simulated *Bz* signal is shown in Figure 14a, while the curves of ∆*Bz* with respect to the number of turns are presented in Figure 14b.

Within the range of 300 to 800 turns for the excitation coil, ∆*Bz* linearly and steadily increases with the number of turns. However, within the range of 800 to 1000 turns, ∆*Bz* shows a slower increase. Therefore, within a certain range of coil turns, increasing the number of turns in the excitation coil leads to higher detection sensitivity of the probe. However, as the number of turns in the excitation coil increases, the growth of detection sensitivity becomes slower. Considering the gain effect of the number of turns of the excitation coil on the detection sensitivity of the probe and the space limitation inside the ACFM probe outer shell, this paper finally uses 0.2 mm thick purple copper enameled wire to wind 800 turns of excitation coils on a U-shaped magnetic core.

#### 4.2.2. Optimization of Detection Coil

**Figure 15:**
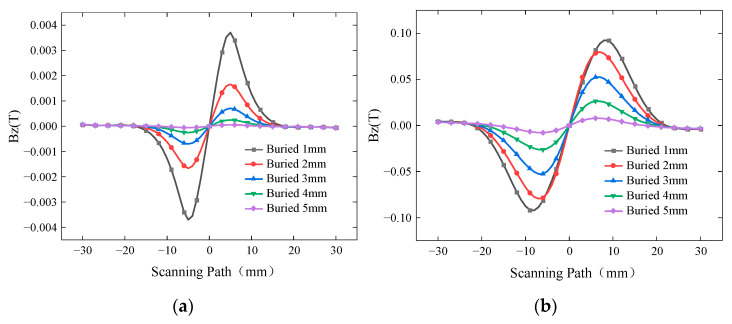
*Bz* signals with or without Magnetic core for different burial depths. (**a**) *Bz* signal without magnetic core. (**b**) *Bz* signal with magnetic core.

**Figure 16:**
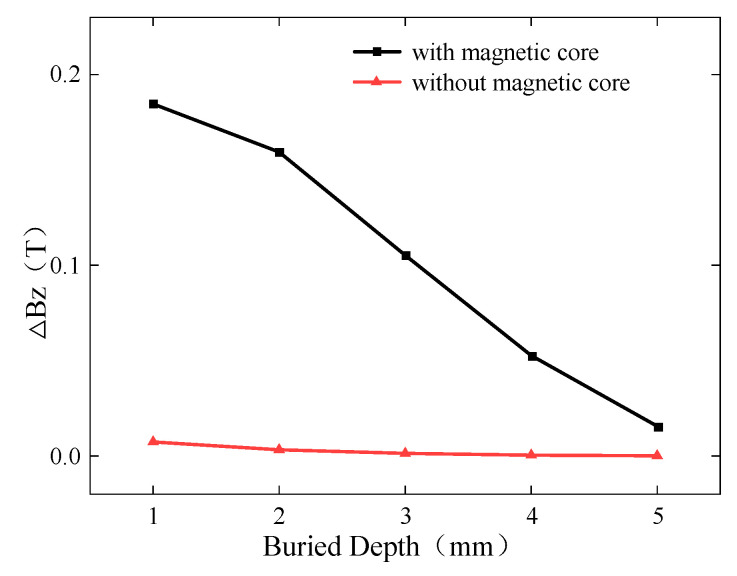
Variation in ∆*Bz* with burial depth for presence or absence of magnetic core.

To improve the detection performance of the ACFM probe, the detection coil was optimized in this study. The manganese-zinc ferrite magnetic core placed at the center of the detection coil not only affects the inductance of the detection coil but also provides magnetic field focusing. To verify the optimization effect of the magnetic core on the detection signal, simulation models were established with and without the magnetic core to detect buried defects at different depths. The simulated *Bz* signal is shown in Figure 15, while the curves of ∆*Bz* with and without the magnetic core are presented in Figure 16.

For buried depths ranging from 1 mm to 5 mm, ∆*Bz* with and without the magnetic core decreases as the depth of the buried defect increases. However, the simulation results with the magnetic core show significantly higher detection sensitivity compared to those without the magnetic core, with the sensitivity being several times higher. Therefore, the optimized design of the detection coil greatly enhances the detection sensitivity of the probe.

### 4.3. Overall Design of the Detection System

**Figure 17:**
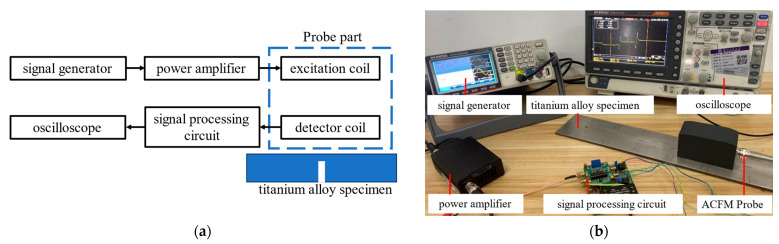
Schematic and diagram of ACFM detection system. (**a**) Schematic of ACFM detection system. (**b**) ACFM detection system diagram.

To investigate the detection performance of the detection probe for buried defects in titanium alloy plates and the characteristics of the signals generated by buried defects, the block diagram and system diagram of the ACFM detection system constructed in this study are shown in Figure 17. The ACFM detection system consists of a signal generator (MFQ-2230M, 2 Channel), a power amplifier (FPA301-20W 5 MHz), an ACFM detection probe, signal processing circuitry, titanium alloy specimens, and an oscilloscope (GDS-2102E). The signal generator outputs an AC sine signal, which is accurately amplified multiple times by the power amplifier and applied to the excitation coil of the ACFM probe. The distorted magnetic field caused by defects is captured by the detection coil, and after amplification, filtering, and demodulation in the signal processing circuitry, the change in the magnetic field with the scanning position is displayed on the oscilloscope.

## 5. Experimental Test and Analysis

### 5.1. Surface Defect Detection Test

#### 5.1.1. Test on Surface Defects of Different Lengths

**Figure 18:**
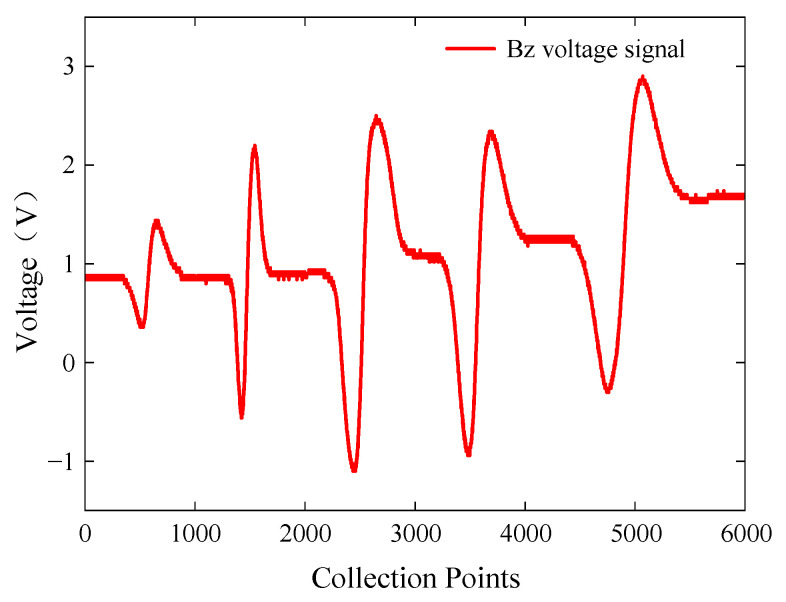
*Bz* voltage signals for surface defects of different lengths.

To investigate the influence of surface defect length on characteristic signals, the cracks on specimen A were detected using the ACFM detection system. Specimen A with cracks was placed with the defective side facing up, and the probe scanned cracks 1 to 5 in a uniform motion. The excitation frequency of the signal generator was set to 10 kHz with a voltage amplitude of 5 V. The extracted Bz voltage signals from the detection coil are shown in Figure 18.

The probe was able to detect surface defects with lengths ranging from 3 mm to 15 mm. However, due to the high precision of the probe and the uneven distribution of materials inside the specimen, the steady-state value of the voltage signals was offset, which also occurred in subsequent experiments. Within the range of defect lengths from 3 mm to 9 mm, the peak-to-peak value of the *Bz* signal increased sharply. Within the range of defect lengths from 9 mm to 15 mm, the peak-to-peak value of the *Bz* signal slowly decreased. The probe showed good detection performance for surface defects of different lengths.

#### 5.1.2. Test on Surface Defects of Different Depths

**Figure 19:**
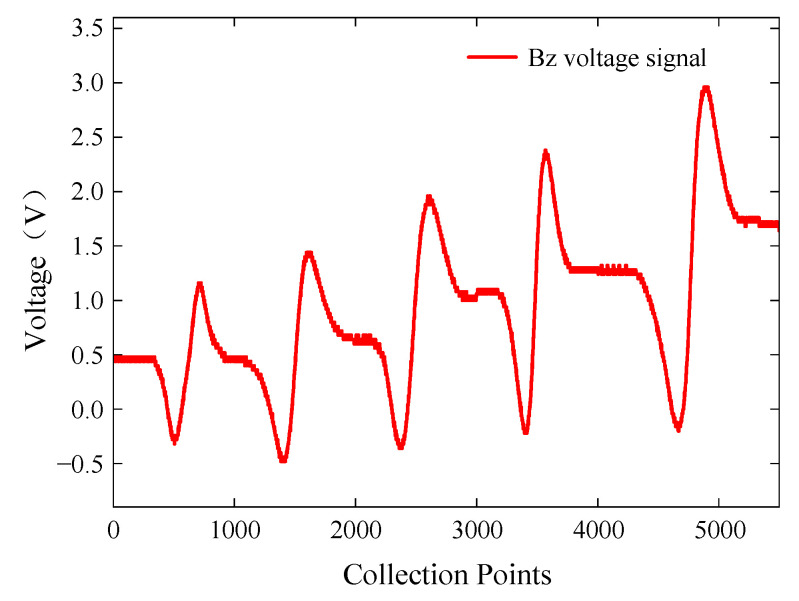
*Bz* voltage signals for surface defects of different depths.

To investigate the influence of surface defect depth on characteristic signals, specimen B with cracks 6 to 10 was placed with the defective side facing up, and the probe scanned the cracks sequentially. The excitation frequency of the signal generator was set to 10 kHz with a voltage amplitude of 5 V. The extracted *Bz* voltage signals from the detection coil are shown in Figure 19.

The probe was able to detect surface defects with depths ranging from 1 mm to 5 mm. Within the range of eddy current penetration depth, the deeper the defect, the greater the induced current density accumulated at the ends of the defect, resulting in a larger magnetic field distortion. Within the range of depths from 1 mm to 5 mm, the peak-to-peak value of the *Bz* signal increased sharply.

### 5.2. Buried Defect Detection Test

#### 5.2.1. Test on Buried Defects of Different Lengths

**Figure 20:**
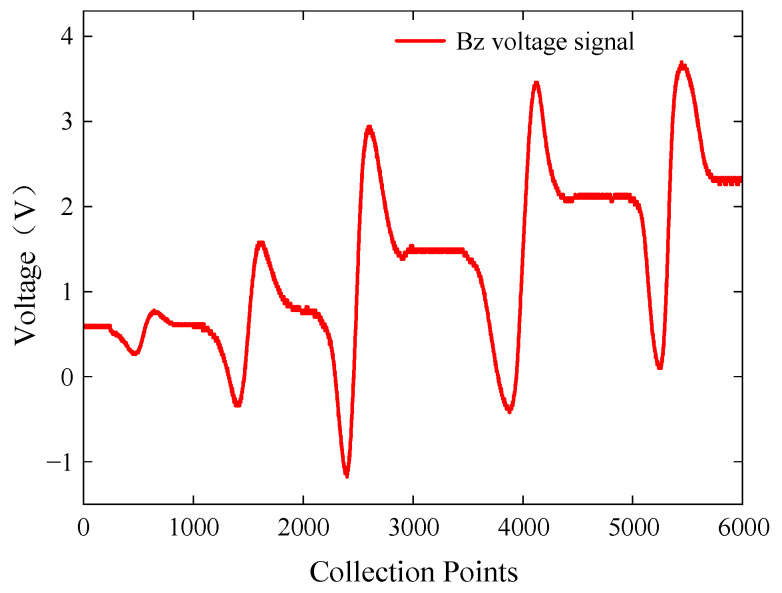
*Bz* voltage signals for buried defects of different lengths.

To investigate the influence of buried defect length on characteristic signals, specimen A was placed with the defective side facing down, and the probe sequentially scanned cracks 1 to 5. In the ACFM method, the scanning direction of the probe greatly affects the detection rate of defects. To ensure effective detection of buried defects, the ACFM probe was aligned parallel to the placement of the defect, and the probe scanned along the direction of the defect. However, in practical testing, the orientation of cracks is not known, and weak detection signals cannot definitively determine the absence of cracks in a particular area. Therefore, to ensure defect detection, multiple directional scans should be performed during the actual testing process. The excitation frequency of the signal generator was set to 7 kHz with a voltage amplitude of 5 V. The extracted *Bz* voltage signals from the detection coil are shown in Figure 20.

The probe was able to detect buried defects with lengths ranging from 3 mm to 15 mm. The *Bz* signal reached its extreme value at the ends of the cracks, and the interval between the signal peaks and valleys generally increased with the increase in defect length. Within the range of buried defect lengths from 3 mm to 9 mm, the peak-to-peak value of the *Bz* signal increased sharply. Within the range of buried defect lengths from 9 mm to 15 mm, the peak-to-peak value of the *Bz* signal showed a decreasing trend. As the crack length increased, more induced current accumulated at the ends of the crack, resulting in a larger magnetic field distortion. However, as the crack length continued to increase, the induced current density distribution in the crack region decreased, leading to a decrease in the peak-to-peak value of the signal.

#### 5.2.2. Test on Buried Defects of Different Depths

**Figure 21:**
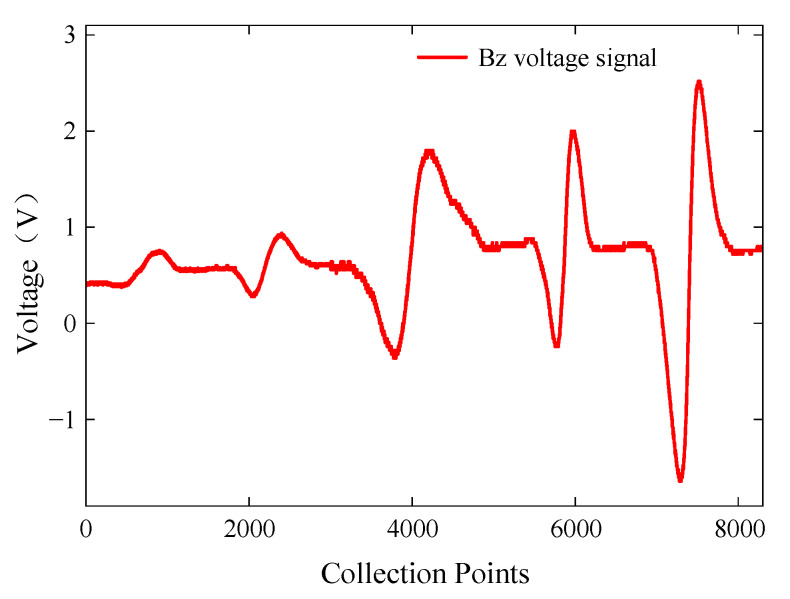
*Bz* voltage signals for buried defects of different depths.

To investigate the influence of buried defect depth on characteristic signals, specimen B with cracks 6 to 10 was placed with the defective side facing down, and the probe scanned the cracks sequentially. The ACFM probe was aligned parallel to the placement of the defects and scanned along the direction of the cracks. The excitation frequency of the signal generator was set to 7 kHz with a voltage amplitude of 5 V. The extracted *Bz* voltage signals from the detection coil are shown in Figure 21.

The probe was able to detect buried defects with depths ranging from 2 mm to 5 mm, while the probe was unable to effectively detect buried defects with a depth of 1 mm. In the actual detection process, defects are often irregular, in the appropriate excitation frequency, in the range of effective penetration depth of the induced current, with a high detection sensitivity of the probe can detect a certain depth range of defects. Within the range of depths from 2 mm to 5 mm, the peak-to-peak value of the *Bz* signal showed an increasing trend. In other words, the deeper the defect, the smaller the attenuation of the magnetic field signal due to the buried depth. Therefore, the peak-to-peak value of the *Bz* signal increases with an increase in the defect depth.

### 5.3. Analysis of Experimental Results

**Figure 22:**
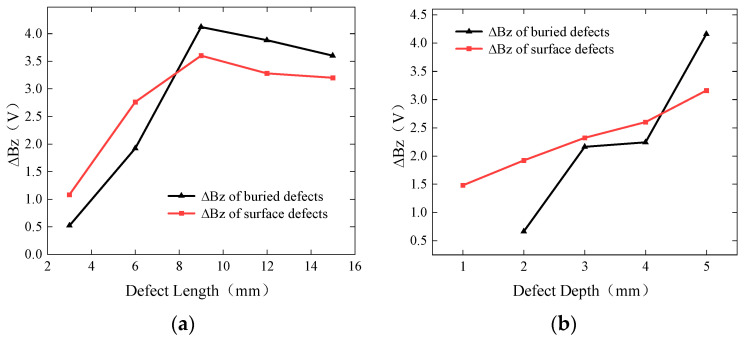
Curve of *Bz* peak-to-peak values with variation in length and depth. (**a**) Curve of *Bz* peak-to-peak values with length variation. (**b**) Curve of *Bz* peak-to-peak values with depth variation.

Based on the detection results of surface defects and buried defects in the titanium alloy plate, curve graphs of the peak-to-peak value of the *Bz* signal against defect length and depth were plotted, as shown in Figure 22. From Figure 22a, as the defect length keeps increasing, the density of induced currents around the defect decreases, resulting in a decrease in the peak-to-peak values of the signals for both surface defects and buried defects with a defect length of 9 mm. It can also be observed that as the defect length increases from 3 mm to 9 mm, the peak-to-peak values of the signals for both surface defects and buried defects increase sharply and reach extreme values. When the defect length is 9 mm, the corresponding peak-to-peak values of the signals are 3.6 V and 4.12 V for surface defects and buried defects, respectively. As the defect length increases from 9 mm to 15 mm, the peak-to-peak values of the signals for both surface defects and buried defects show a decreasing trend. When the defect length increases from 3 mm to 6 mm, the *Bz* signal peak-to-peak values for surface defects and buried defects increase by 156% and 269%, respectively, indicating that the probe has high sensitivity to changes in defect length. The ACFM probe is able to effectively detect shallow surface microcracks with a length of 3 mm and depth of 4 mm, as well as buried microcracks with a length of 3 mm and a burial depth of 2 mm.

From Figure 22b, it can be observed that as the defect depth increases from 1 mm to 5 mm, the peak-to-peak values of the signals for both surface defects and buried defects show an increasing trend. When the defect depth is 5 mm, the peak-to-peak values of the signals for surface defects and buried defects are 3.16 V and 4.16 V, respectively. When the defect depth increases from 2 mm to 3 mm, the *Bz* signal peaks.

## 6. Conclusions

This paper addresses the issue of buried defects in titanium alloy aircraft skin and establishes an ACFM simulation model for detecting buried defects in titanium alloy specimens. A detection probe based on the alternating current electromagnetic field detection method is designed. The relationship between the characteristics of the buried defect and the excitation frequency is investigated, and an ACFM detection system is constructed. The feasibility of the detection probe for detecting buried defects in titanium alloy aircraft skin is verified through simulation and experimentation, leading to the following conclusions:

(1) Under the excitation of the alternating current electromagnetic field, the characteristics of the buried defect signal are consistent with those of surface defects. The *Bx* signal exhibits a valley in the middle of the crack and peaks at both ends. The *Bz* signal displays peaks and valleys at the ends of the crack. The alternating current electromagnetic field detection method can be used for detecting buried defects in aerospace titanium alloy specimens. The *Bx* signal can indicate the depth information of the buried defect, while the *Bz* signal can indicate the length information of the buried defect.
(2) The influence of buried defect length and depth on the *Bz* signal is investigated. As the length of the buried defect increases, the peak-to-peak value of the *Bz* signal follows an initially increasing and then decreasing pattern. As the depth of the buried defect increases, the peak-to-peak value of the *Bz* signal shows a linear increase The experimental results are in good agreement with the simulation results. The ACFM probe exhibits high sensitivity to changes in the length and depth of buried defects, allowing for preliminary determination of the length and depth information of buried defects.
(3) Surface defect detection results of titanium alloy specimens based on the ACFM method indicate that the probe can effectively detect small cracks with a length of 3 mm and a depth of 4 mm, as well as deep defects with a length of 10 mm and a depth of 5 mm, with corresponding peak-to-peak values of 1.08 V and 3.16 V, respectively. Buried defect detection results of titanium alloy specimens indicate that the probe can effectively detect small cracks with a length of 3 mm and a burial depth of 2 mm, as well as deep defects with a length of 10 mm and a burial depth of 4 mm, with corresponding peak-to-peak values of 0.52 V and 0.66 V, respectively.

The ACFM detection probe and method studied in this paper can effectively detect small buried defects in thin-walled titanium alloy specimens, providing a basis for detecting buried defects in titanium alloy aircraft skin in the future.

## Figures and Tables

**Table 1:** ACFM Simulation Model Dimensional Parameters.

Simulation Model Dimensional Parameters	Value (mm)
Titanium alloy plate length × width × thickness	140 × 80 × 6
U-shaped magnetic core length × width × height × thickness	54 × 24 × 30 × 12
Buried defect length × width × depth	10 × 0.5 × 4

**Table 2:** ACFM Simulation Model Material Parameters.

Model Name	Material	Relative Permeability	Conductivity (S·m^−1^)
Plate	Titanium alloy	1	6 × 10^5^
Excitation coil	Copper	1	5.998 × 10^7^
U-shaped magnetic core	Manganese zinc ferrite	2400	0.1

## Data Availability

Data is unavailable due to privacy reasons.
